# Effectiveness of eHealth Interventions in Improving Medication Adherence for Patients With Chronic Obstructive Pulmonary Disease or Asthma: Systematic Review

**DOI:** 10.2196/29475

**Published:** 2021-07-27

**Authors:** Mieke H J Schulte, Jiska J Aardoom, Lisa Loheide-Niesmann, Leonie L L Verstraete, Hans C Ossebaard, Heleen Riper

**Affiliations:** 1 Department of Clinical, Neuro- and Developmental Psychology Vrije Universiteit Amsterdam Netherlands; 2 Department of Public Health and Primary Care Leiden University Medical Center Leiden Netherlands; 3 National eHealth Living Lab Leiden Netherlands; 4 Behavioural Science Institute Radboud University Nijmegen Netherlands; 5 Dutch National Healthcare Institute Diemen Netherlands; 6 Department of Psychiatry Amsterdam University Medical Center (VUmc) Vrije Universiteit Amsterdam Netherlands; 7 GGZ inGeest Specialized Mental Health Care Research and Innovation Amsterdam Netherlands

**Keywords:** chronic obstructive pulmonary disease, asthma, medication adherence, exercise adherence, treatment adherence, eHealth, systematic review, COPD, adherence, exercise, treatment, review

## Abstract

**Background:**

Poor treatment adherence in patients with chronic obstructive pulmonary disease (COPD) or asthma is a global public health concern with severe consequences in terms of patient health and societal costs. A potentially promising tool for addressing poor compliance is eHealth.

**Objective:**

This review investigates the effects of eHealth interventions on medication adherence in patients with COPD or asthma.

**Methods:**

A systematic literature search was conducted in the databases of Cochrane Library, PsycINFO, PubMed, and Embase for studies with publication dates between January 1, 2000, and October 29, 2020. We selected randomized controlled trials targeting adult patients with COPD or asthma, which evaluated the effectiveness of an eHealth intervention on medication adherence. The risk of bias in the included studies was examined using the Cochrane Collaboration’s risk of bias tool. The results were narratively reviewed.

**Results:**

In total, six studies focusing on COPD and seven focusing on asthma were analyzed. Interventions were mostly internet-based or telephone-based, and could entail telemonitoring of symptoms and medication adherence, education, counseling, consultations, and self-support modules. Control groups mostly comprised usual care conditions, whereas a small number of studies used a face-to-face intervention or waiting list as the control condition. For COPD, the majority of eHealth interventions were investigated as an add-on to usual care (5/6 studies), whereas for asthma the majority of interventions were investigated as a standalone intervention (5/7 studies). Regarding eHealth interventions targeting medication adherence for COPD, two studies reported nonsignificant effects, one study found a significant effect in comparison to usual care, and three reported mixed results. Of the seven studies that investigated eHealth interventions targeting medication adherence in asthma, three studies found significant effects, two reported nonsignificant effects, and two reported mixed effects.

**Conclusions:**

The mixed results on the effectiveness of eHealth interventions in improving treatment adherence for asthma and COPD are presumably related to the type, context, and intensity of the interventions, as well as to differences in the operationalization and measurement of adherence outcomes. Much remains to be learned about the potential of eHealth to optimize treatment adherence in COPD and asthma.

## Introduction

With a global prevalence of over 299 million people living with chronic obstructive pulmonary disease (COPD) and almost 273 million people living with asthma in 2017 [1], COPD and asthma are common chronic lung diseases. They are a worldwide public health concern and they increasingly affect the lives of patients due to climate change and pollution [2]. The clinical and economic burden of asthma and COPD have been widely established [3]. Both these respiratory diseases are typically treated and managed with drug therapies, often in the form of daily inhaled medication. Full adherence is important for optimal management and treatment of COPD and asthma [4-6]. This is especially the case when patients become more vulnerable, such as during environmental disruptions or the current COVID-19 pandemic [7].

Unfortunately, adherence to treatment regimens for COPD and asthma is often poor. Adherence to inhaled corticosteroids (ICS) in adult patients with asthma is estimated to range from 22% to 63% [8]. Medication adherence in patients with COPD reportedly ranges from 0.3% to 68%, depending on the type and combination of medications [9]. Adherence is a complex, multifaceted concept, including many potential contributing factors, which can be medication-related (eg, side effects) or patient-related (eg, forgetfulness, medication beliefs) [10]. Poor adherence can severely impact patients’ health outcomes, with consequences including an increased risk of mortality and exacerbations, as well as diminished disease control and quality of life [5]. Poor adherence has furthermore been associated with higher health care utilization and costs [5,6]. Therefore, there is an urgent need for interventions that can improve treatment adherence in individuals with COPD or asthma.

A recent Cochrane review comprising 28 randomized controlled trials reported positive effects of various interventions to improve adherence to ICS in asthma in comparison to usual care [11]. After approximately 71 weeks of follow-up, a 20% improvement was achieved for people who were given education about adherence (20 trials) or who were provided with electronic monitoring or reminders to use their inhaler (11 trials). Another review investigated the effect of interventions to improve medication adherence in COPD [12]. Overall, five of the seven studies reported significant improvements in adherence. Effective strategies involved brief counseling, monitoring, and feedback on adherence through electronic medication delivery devices, as well as multicomponent interventions including education, self-management, motivational interviewing, and extra support (eg, clinic visits, phone calls) by health care professionals. Whether such strategies produced effects of similar magnitudes remains unclear.

Increasingly, eHealth is being used in the provision of health care services such as patient communication, monitoring, and education. In general, eHealth can be an effective tool to address poor treatment adherence in patients with chronic diseases, as indicated by the results of numerous studies focusing on different target populations [13-17]. However, to our knowledge, no systematic review or meta-analysis has yet been performed investigating the effects of eHealth interventions on adherence specifically for patients with COPD and only limited research has focused on youth or adult asthma populations. Bonini [18] conducted a systematic review of the literature published in 2016 that assessed the effects of eHealth on asthma management, incorporating multiple components including medication adherence in children and adults. The findings suggested an overall beneficial effect of eHealth on asthma control and management, whereby eHealth included mobile health systems (mHealth), telemedicine, electronic health records, and digital app interventions. A recent meta-analysis investigated the effect of eHealth on ICS adherence in patients with asthma (including both children and adults), comprising 15 randomized controlled trials with a total of 13,907 participants. Compared with usual care, a small but significant overall effect of eHealth interventions was observed. In addition, a pooled analysis of four studies provided evidence for the superiority of mHealth interventions such as SMS text messages and audiovisual reminders as compared to usual care [19].

The aim of our study was to systematically review the effectiveness of eHealth interventions in improving medication adherence in adult patients with COPD or asthma. In this review, the results were presented separately for asthma and COPD in order to enable investigation of potential differences between the diseases, as well as to allow for potential nuance in terms of the effectiveness of specific eHealth interventions for the two diseases separately.

## Methods

No review protocol was made beforehand and the review was not registered in any database or registry. The PRISMA (Preferred Reporting Items for Systematic Reviews and Meta-Analyses) checklist can be found in Multimedia Appendix 1.

### Search Strategy

Our search strategy was part of a broader search performed in a research project on the role of eHealth in treatment adherence in chronic lung disease, including obstructive sleep apnea (OSA), asthma, and COPD. The results regarding OSA have been published elsewhere [20]. The search was conducted in the electronic databases of the Cochrane Library (Wiley), PsycINFO (EBSCO), PubMed, and Embase. The search results were limited to available full-text articles in English or Dutch with publication dates from January 1, 2000, to October 29, 2020. Numerous terms related to eHealth technology, patient adherence, and the target populations (asthma, COPD) were combined, using both free-text and index terms (for the full search string, see Multimedia Appendix 2). In addition, reference lists of the included studies, as well as relevant systematic reviews, were checked for potentially relevant additional studies.

### Study Selection and Data Extraction

Inclusion criteria were as follows: (1) The target population comprised patients aged ≥18 years with COPD or asthma. (2) One or more main component(s) of the intervention were delivered by eHealth technology, or an eHealth component was investigated as an add-on intervention to usual care. The criteria to qualify as an eHealth intervention were that (A) the intervention was delivered via information and communications technology such as telephone calls, telemedicine (eg, videoconferencing), websites, smartphone apps, or SMS text messages; and (B) the intervention was delivered independently of time and place (eg, videos delivered in face-to-face sessions were not considered eligible). (3) Intervention effects were compared to a control group, with exclusion of control conditions containing the same eHealth component as the experimental condition. (4) Outcomes were assessed in terms of at least one quantitative measure of adherence to the medical treatment—that is, to oral or inhaler medication. (5) Adherence measures were compared statistically between study conditions. (6) The study design was a randomized controlled trial.

All titles and abstracts were independently screened by two reviewers (JA and LL: January 1, 2000, through March 20, 2018; MS and LV: March 21, 2018, through October 29, 2020). Subsequently, the full-text articles of the selected papers were screened to determine eligibility for this review. Covidence software was used to manage the screening process and the risk-of-bias assessments. Data on study reference, design, population, interventions, outcomes, and results were extracted by means of a data extraction form in an Excel spreadsheet by JA (January 1, 2000, through March 20, 2018) and MS (March 21, 2018, through October 29, 2020).

### Quality Assessment

The Cochrane Collaboration’s risk-of-bias tool [21] was used to assess the quality of all included studies. Two reviewers (JA and LL or MS and LV, depending on publication date; see above) independently evaluated the following dimensions of risk of bias: (1) adequacy of random sequence generation, (2) adequacy of concealment of allocation sequence to personnel, (3) blinding of study participants and personnel, (4) blinding of outcome assessors, (5) adequacy of handling of incomplete outcome data, and (6) selective outcome reporting. Each study was rated per dimension as “low risk,” “high risk,” or “unclear risk.” Disagreements between reviewers were resolved by discussion.

### Data Analysis

Due to the limited number of available studies and the heterogeneity of the studies in terms of designs and characteristics, as well as the assessments and operationalizations of medication adherence, the results were narratively reviewed and no meta-analysis has been performed.

## Results

### Search and Screening

**Figure 1:**
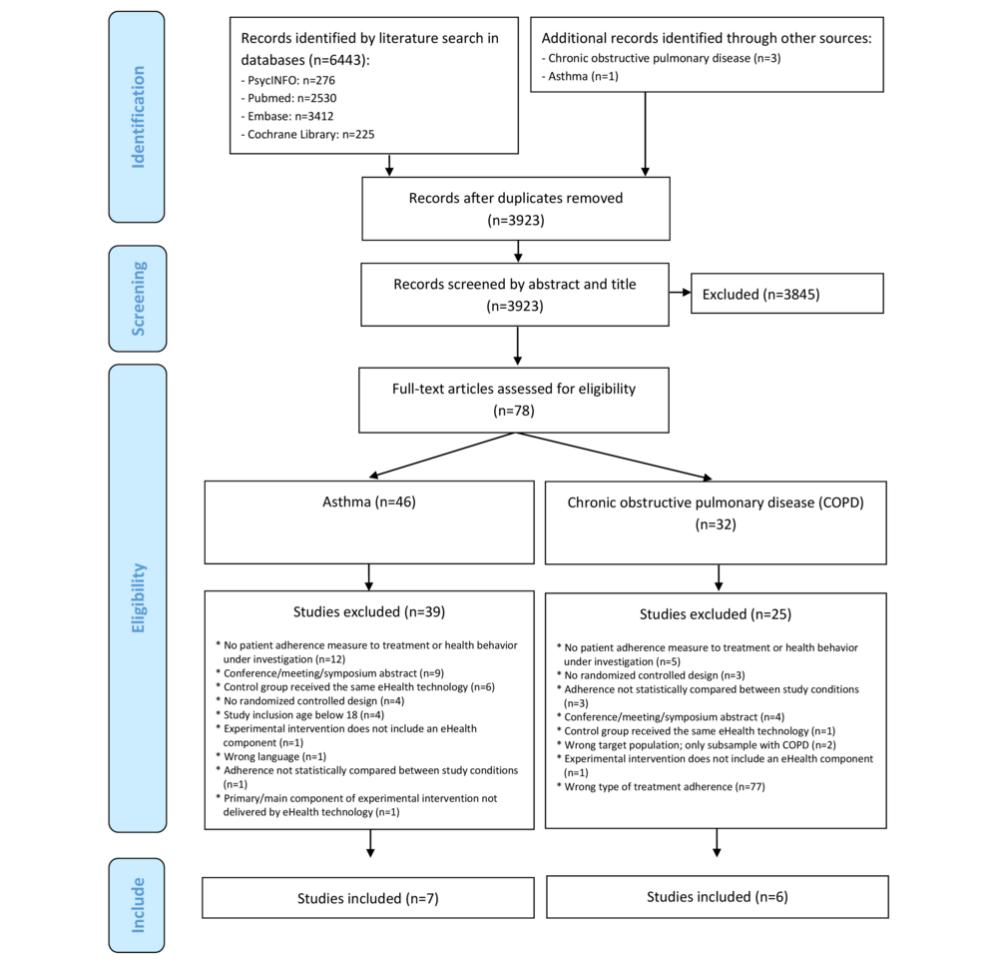
PRISMA flowchart describing study identification and selection process. PRISMA: Preferred Reporting Items for Systematic Reviews and Meta-Analyses.

Figure 1 depicts the PRISMA flow diagram of study identification and selection. The pooled systematic search resulted in a total of 6447 potentially relevant articles covering COPD or asthma. After removal of 2520 duplicates, a total of 3923 articles were selected for title and abstract screening. A total of 78 studies were then selected for full-text screening. Of these, 32 targeted COPD and 46 targeted asthma. Full-text screening of the eligibility criteria eventually led to the inclusion of 6 studies targeting COPD and 7 studies targeting asthma.

### Results for COPD

#### Study Characteristics

Multimedia Appendix 3 provides an overview of study and intervention characteristics; of the six included studies, two interventions were internet-based and four were telephone-based. Interventions involved the telemonitoring of symptoms and adherence, education and counseling (eg, knowledge of the disease, smoking cessation, inhaler techniques), and self-support modules (eg, to help patients identify disease exacerbations or to support psychological well-being). Medication adherence was a primary outcome in three studies and a secondary outcome in three others. The studies were conducted in Europe, China, and New Zealand.

#### Quality Assessment

**Figure 2:**
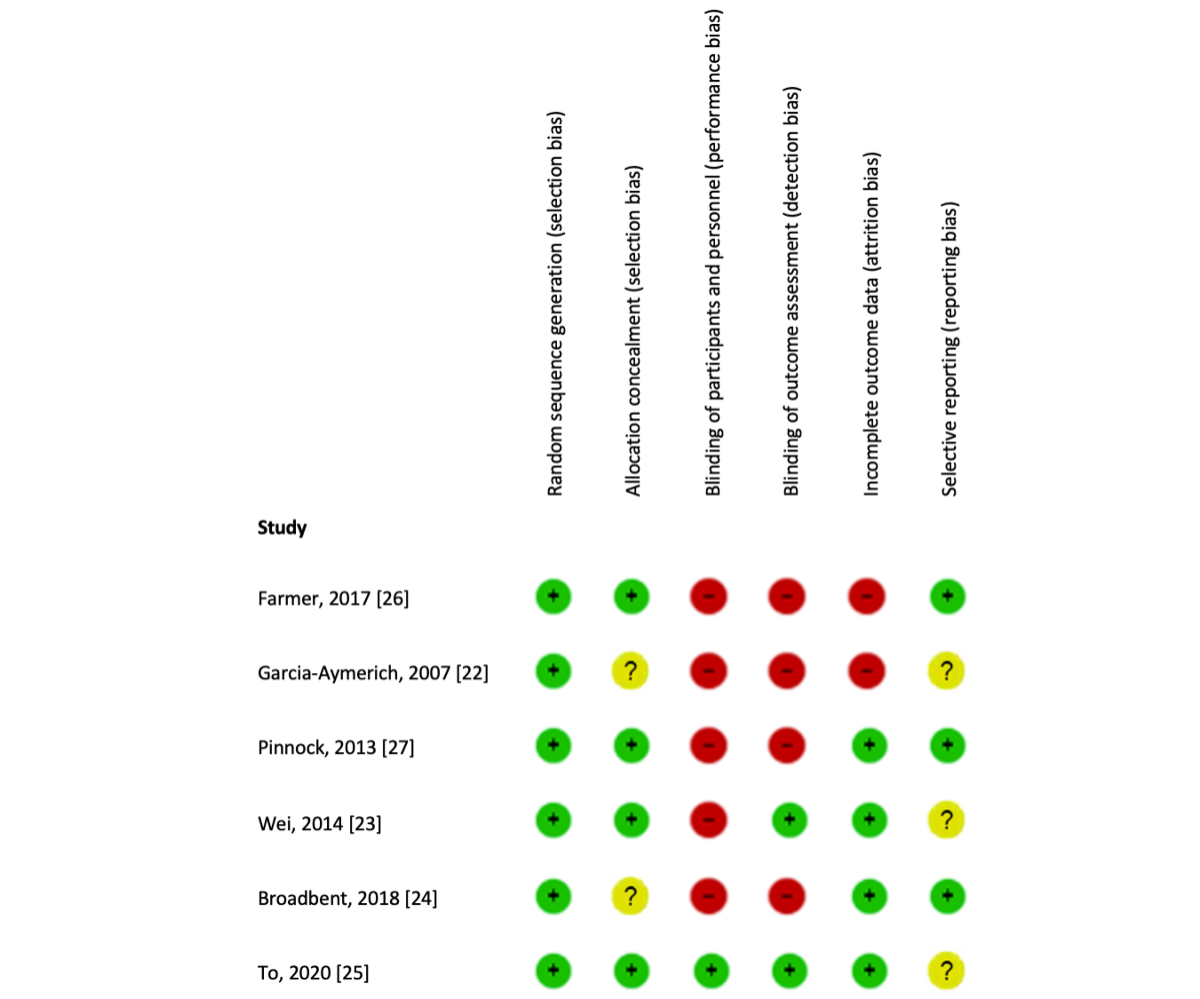
Risk of bias analysis on individual studies investigating effectiveness of eHealth interventions on medication adherence in patients with chronic obstructive pulmonary disease.

Figure 2 presents the results of the risk-of-bias assessment for each study separately. None of the studies were rated as having low risk of bias on all six dimensions. The majority of studies had a high risk of performance bias (n=5) and detection bias (n=4). These were due to the lack of blinding and participants self-reporting their medication adherence while being aware of their allocated study condition. Studies with a high risk of attrition bias (n=2) generally did not analyze the data according to an intention-to-treat design, thus excluding participants who did not adhere to the intervention or were lost to follow-up.

#### Effects of eHealth Interventions on Medication Adherence

The effectiveness of the interventions is shown in Table 1. Of the six included studies, four studies focusing on medication adherence in COPD demonstrated the eHealth intervention to be more effective than care as usual in terms of improving medication adherence on at least one of the operationalizations of adherence [22-25]. However, three of these four studies reported nonsignificant effects when other operationalizations of medication adherence were used [22,24,25]. In addition, two studies found no differences between participants who received the eHealth intervention (ie, online monitoring and as-needed telephone contact) supplemental to care as usual, or care as usual only [26,27]. Both of these studies used the self-report Medication Adherence Rating Scale (MARS).

Telephone-based pharmaceutical care including education and counseling was found to be effective as compared to care as usual in terms of adherence operationalized as pill count [23]. The three studies that reported mixed results all investigated the effect of an eHealth intervention as an add-on to care as usual. These studies differed regarding the type of intervention and the technology used, as well as how medication adherence was operationalized. In one study, telephone-based integrated care resulted in significant effects in terms of the percentages of self-reported medication inhaler adherence and observed correct inhaler maneuvers, whereas no effects were found for the percentages of self-reported oral medication adherence [22]. In another study, telephone-based telemonitoring did not result in significant effects on adherence to the medication regimen, but there were significant effects on the percentages of people adhering at least 80% to the regimen; in both those operationalizations, adherence was measured objectively by an administration tracker attached to the device [25]. In the last study, telemonitoring and treatment reminders delivered by an internet-linked robot resulted in significant results when medication adherence was measured with a self-reported questionnaire, but nonsignificant results when the percentages of medication adherence were measured objectively by an administration tracker attached to the device [24].

### Results for Asthma

#### Study Characteristics

Multimedia Appendix 3 presents the details on study and intervention characteristics. Of the seven included studies focusing on asthma, six interventions were telephone-based [28-33] and one was internet-based [34]. Interventions involved monitoring and management of medication, including reminders for intake and refills, as well as pharmacist consultations. Control conditions included, among others, asthma education, monitoring and treatment by a general practitioner or specialist, and inhaler use tracking. Study periods ranged from 2.5 to 18 months. Overall, five studies included medication adherence as a primary outcome. Most studies were conducted in the United States (n=4).

#### Quality Assessment

**Figure 3:**
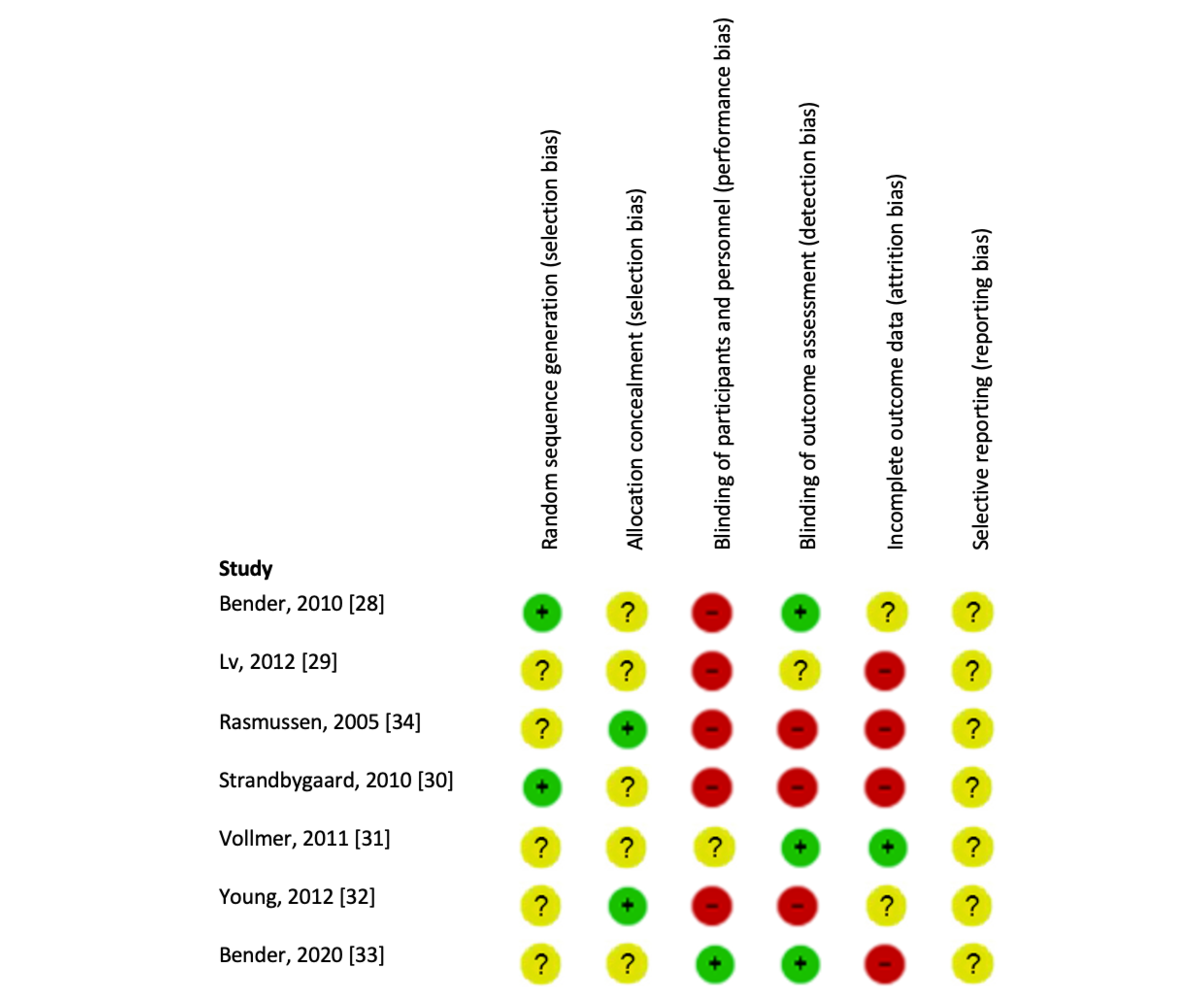
Risk of bias analysis on individual studies investigating effectiveness of eHealth interventions on medication adherence in patients with asthma.

Figure 3 presents the results of the risk-of-bias assessment. None of the studies were rated as having low risk of bias on all six dimensions. A substantial number of studies had a high risk of performance bias (n=5) or detection bias (n=3), due to the lack of blinding. Most studies also had a high risk of attrition bias (n=4), mainly because they did not analyze the data in an intention-to-treat design.

#### Effects of eHealth Interventions on Medication Adherence

The effectiveness of the interventions is displayed in Table 1. We determined that five of the seven included studies on medication adherence in asthma reported significant effects on at least one of the operationalizations of medication adherence compared to control conditions [28,30,31,33,34]. However, at the same time, two of these five studies reported nonsignificant effects when other operationalizations of medication adherence were used [30,33]. In addition, two studies reported nonsignificant effects [29,32]. In two studies, a standalone eHealth intervention involving an interactive voice response system for monitoring, medication reminders, and education was found to significantly increase self-reported medication adherence [28,31]. One of these studies operationalized medication adherence on the basis of self-report [31], whereas the other tracked inhaler use or assessed inhaler weight [28]. In another study, a guided standalone internet-based monitoring and management tool was found to significantly increase self-reported medication adherence as compared to a control group receiving monitoring and treatment by a general practitioner or specialist [34]. Another study also investigating the effect of SMS text message medication reminders as an add-on to care as usual reported mixed results depending on how adherence was operationalized [30]. Compared to care as usual, there was a significant improvement in the percentage of inhaler use, but not in the percentage of participants collecting their medication refills, as recorded in pharmacy reports. Another study also investigated the effect of a standalone monitoring and management support tool, but provided via SMS text message or telephone interactive voice response or via email [33]; the results were mixed depending on the operationalization of medication adherence and the group comparisons. Comparing the combined intervention groups with care as usual, the study found no significant effects on self-reported medication adherence. When the intervention groups were compared, no significant effect was found on self-reported use of reliever medication or on the asthma medication ratio, but a greater increase in self-reported use of controller medication was found in the SMS text message/telephone group as compared to the email group. Telephone-based pharmacist consultations as an add-on to care as usual did not result in a significant increase in self-reported adherence rates compared with care as usual [32]. Lv and colleagues [29] investigated the effect of a standalone eHealth intervention using SMS reminders about asthma management; they reported nonsignificant effects in comparison with care as usual or with verbal or written asthma education. Medication adherence was defined as the percentage of participants that were adherent, although the exact operationalization was unclear.

**Table 1:** Effects of eHealth interventions for medication adherence in chronic obstructive pulmonary disease and asthma: study results^a^.

Condition; reference and study design; and outcome operationalization			Outcome measure	Between-group results	Between-group statistic (*P* value)	Study quality (number of dimensions unclear/low/high risk)^b^
**Chronic obstructive pulmonary disease**						
	**Farmer, 2017 [26], add-on to care as usual**				.77	0/3/3
		Medication adherence	Medication Adherence Rate Scale	Difference in score from baseline to 12 months. TG: 0.17 (2.47) vs CG: 0.33 (3.65).		
	**Garcia-Aymerich, 2007 [22], add-on to care as usual**					2/1/3
		Percentage of oral medication adherence	Medication Adherence Scale	12-month results. TG: 90% vs CG: 85%	.57	
		Percentage of inhaler medication adherence	Inhaler Adherence Scale	12-month results. TG: 71% vs CG: 37%	.009	
		Percentage of correct inhaler maneuvers	Observed inhaler skills	12-month results. TG: 86% vs CG: 24%	.001	
	**Pinnock, 2013 [27], add-on to care as usual**					0/4/2
		Medication adherence	Medication Adherence Rate Scale	12-month results. TG: 24.0 (1.7) vs CG: 23.7 (1.9)	.05	
	**Wei, 2014 [23], standalone**					1/4/1
		Percentage of medication adherence	Pill count	12-month results. TG: 66.5 (8.6) vs CG: 54.4 (12.5)	.04	
	**Broadbent, 2018 [24], add-on to care as usual**					1/3/2
		Percentage of medication adherence	Administration tracker on device	TG: 48.5% vs CG: 29.5%	.03	
		Medication adherence	Medication Adherence Rate Scale	Difference in score from baseline to 4 months. TG: 1.63 (0.56) vs CG: 0.12 (0.55)	.06	
	**To, 2020 [25], add-on to care as usual**					1/5/0
		Medication regimen adherence	Ratio number of doses taken (administration tracker) / number of doses prescribed	2-month results. TG: 99.8 (15.0) vs CG: 92.7 (30.0)	.12	
		Percentage of ≥80% adherence	Ratio number of doses taken (administration tracker)/number of doses prescribed	2-month results. TG: 85.7 vs CG: 71.4	.02	
		Correct inhaler technique	Inhaler use checklist	2-month results. TG: 91.9 (7.8) vs CG: 79.9 (17.1)	.002	
**Asthma**						
	**Bender, 2010 [28], standalone**					3/2/1
		Percentage of inhaled corticosteroids adherence (number of taken puff/number of prescribed puffs)	Electronic tracking device	CG: 64.5 (17.2) vs TG: 49.1 (16.8)	.003	
	**Lv, 2012 [29], add-on to care as usual**					4/0/2
		Percentage of adherers	Not specified	TG: 80 vs CG1: 74.1 vs CG2: 50	.11	
	**Rasmussen, 2005 [34], standalone**					2/1/3
		Percentage of inhaled corticosteroids adherers	Self-reported as almost always taking inhaled corticosteroids	TG: 87 vs CG2: 54	<.001	
		Percentage of inhaled corticosteroids adherers	Self-reported as almost always taking inhaled corticosteroids	CG1: 79 vs CG2: 54	<.001	
	**Strandbygaard, 2010 [30], add-on to care as usual**					2/1/3
		Percentage of inhaled corticosteroids adherence	Administration tracker on device	TG: 81.5 vs CG: 70.1	.02	
		Percentage collecting medication	Pharmacy reports	TG: 64.3 vs CG: 66.7	.69	
		Number of days until collecting medication	Pharmacy reports	TG: 32 (13-50) vs CG: 29 (13-49)^c^	.56	
	**Vollmer, 2011 [31], standalone**				.002	4/2/0
		Change in medication adherence	Difference in modified medication possession ratio	TG: 0.40 (0.32) vs CG: –0.04 (0.24)		
	**Young, 2012 [32], standalone**				.07	3/1/2
		Percentage of low adherers	Morisky Medication Adherence Scale	TG: 47.0 vs CG: 26.0		
	**Bender, 2020 [33], standalone**					3/2/1
		Reliever medication use	Number of beta-agonist canisters dispensed	6- to 18-month results. CG: 4.71 (0.23) vs TG1+TG2: 4.96 (0.16)	.19	
		Reliever medication use	Number of beta-agonist canisters dispensed	6- to 18-month results: TG1: 5.15 (0.27) vs TG2: 4.76 (0.19)	.72	
		Controller medication use	Number of inhaled corticosteroids canisters dispensed	6- to 18-month results: CG: 6.43 (0.26) vs TG1+TG2: 6.70 (0.20)	.19	
		Controller medication use	Number of inhaled corticosteroids canisters dispensed	6- to 18-month results: TG1: 6.85 (0.31) vs TG2: 6.55 (0.24)	.03	
		Ratio used medication	Asthma medication ratio	6- to 18-month results: CG: 0.58 (0.01) vs TG1+TG2: 0.57 (0.01)	.99	
		Ratio used medication	Asthma medication ratio	6- to 18-month results. TG1: 0.57 (0.01) vs TG2: 0.58 (0.01)	.05	

^a^CG: control group; TG: treatment group.

^b^Risk of bias according to six dimensions of the Cochrane Collaboration’s risk of bias tool.

^c^Values represent means (ranges).

## Discussion

### General Discussion

This review investigated the effects of eHealth interventions in improving adherence to medication treatment by patients with COPD or asthma. In general, mixed results were found and no definite conclusions could be drawn.

The mixed results of the current review may be explained by differences in study design, type and intensity of eHealth interventions, type of control condition, and assessment and operationalization of outcome measures. Previous reviews and meta-analyses have demonstrated small but significant effects of eHealth interventions in improving medication adherence for patients with asthma [18,19], as well as for more diverse populations of chronically ill patients [13] and individuals with long-term medication [35]. The latter two studies found positive effects in 66% and 59% of the studies, respectively. In line with our rather inconclusive and mixed findings, all of the abovementioned overview studies highlighted the considerable amount of heterogeneity among study designs and outcomes, and the limited number of high-quality studies conducted.

Differences in operationalization may have contributed to the discrepant results, in that self-report questionnaires such as the MARS [36] might be less sensitive in detecting actual changes in treatment adherence than more direct adherence assessments such as pill counts or inhaler use tracking devices. Indeed, such variations in objective and subjective measurements of medication adherence have been widely reported in the literature [37,38]. To the best of our knowledge, this is the first systematic review to investigate the effects of eHealth interventions on medication adherence in adult patients with COPD or asthma. Our findings are limited by the small number of included studies and considerable heterogeneity regarding different study aspects. This challenges the interpretation of eHealth intervention effects in terms of medication adherence for patients with asthma and COPD.

### Future Research Directions and Recommendations

Given the limited number of high-quality studies, more studies that minimize potential bias risks are needed to create a more substantial and reliable body of research on the effectiveness of eHealth interventions to improve treatment adherence in COPD and asthma. Furthermore, as a wide variety of outcome measures have been used, future studies could benefit from standardizing measures with respect to adherence outcome. In addition, standardizing the operationalizations of such outcome measures and reporting effect sizes instead of mere statistics in terms of significance could potentially lead to more clear-cut results. Future research would benefit from studies with sufficient statistical power. This would also allow for subgroup analyses, which could provide more insight into what types, intensities, and components of interventions might be more effective for different subgroups, ultimately leading to more personalized or tailor-made treatments. Preliminary research suggests that increased adherence can improve patient outcomes as well as reduce health care costs [5,39]. However, more research is needed to elucidate the cost- effectiveness of eHealth interventions targeting adherence in comparison to usual care. Finally, future studies of eHealth interventions should therefore incorporate cost-effectiveness analyses to elucidate effects in relation to costs as compared with usual care.

### Conclusion

No firm conclusion can be drawn due to the small numbers of studies and their heterogeneous results. Much remains to be learned about the potential of eHealth in optimizing treatment adherence in COPD and asthma—for example, in terms of what types and intensities of eHealth intervention components are effective for what types of individuals.

## Appendix

Multimedia Appendix 1 PRISMA 2020 checklist.

Multimedia Appendix 2 Search string.

Multimedia Appendix 3 Study and intervention characteristics.

## Abbreviations

COPD: chronic obstructive pulmonary disease
ICS: inhaled corticosteroids
MARS: Medication Adherence Rating Scale
OSA: obstructive sleep apnea
PRISMA: Preferred Reporting Items for Systematic Reviews and Meta-Analyses
