# Supplementary material for: Effectiveness of eHealth Interventions in Improving Medication Adherence for Patients With Chronic Obstructive Pulmonary Disease or Asthma: Systematic Review
Source: J Med Internet Res. 2021 Jul 27;23(7):e29475. doi: 10.2196/29475 (PMC8403699; doi:10.2196/29475)
Supplement: Multimedia Appendix 2 [file jmir_v23i7e29475_app2.docx]

*Search conducted on October 29, 2020*

PsycINFO (EBSCO)

(DE "Compliance" OR DE "Treatment Compliance" OR DE “Treatment dropouts” OR TX(“fidelity” OR "complian*" OR "non-complian*" OR "noncomplian*" OR "adheren*" OR "non-adheren*" OR "nonadheren*" OR “dropout*” OR “drop-out*” OR “no-show*” OR “noshow*” OR “attend*” OR “non-attend*” OR “nonattend*” OR “absence*” OR “absent*” OR “non-appear*” OR “nonappear*”)) AND (DE "Computer Assisted Therapy" OR DE "Telecommunications Media" OR DE "Electronic Communication" OR DE "Online Social Networks" OR DE "Online Therapy" OR DE "Social Media" OR DE "Telemedicine" OR DE "Text Messaging" OR DE “Computer Mediated Communication” OR DE “Teleconferencing” OR DE “mobile devices” OR DE “communications media” OR DE “cellular phones” OR DE “Internet” OR DE “technology” OR DE “information technology” OR DE “virtual reality” OR DE “computer applications” OR TI(“Internet*” OR “Web*” OR “Online*” OR “tele*” OR “electronic*” OR “video*” OR “device*” OR “digital*” OR “software*” OR “mobile*” OR “technolog*” OR “e-health” OR “ehealth” OR “computer*” OR “e-treat*” OR “e-therap*” OR “mhealth” OR “m-health” OR “distance counsel*” OR “cybercounsel*” OR “cyber-counsel*” OR “cyber-treat*” OR “text-messag*” OR “textmessag*” OR “text messag*” OR “SMS*” OR “texting*” OR “short message service*” OR “smartphone*” OR “cell-phone*” OR “cellphone*” OR “cellular phone*” OR “blended*” OR “handheld device*” OR “hand held device*” OR “iPad*” OR “iPhone*” OR “email*” OR “e-mail*” OR “sensor*” OR “wearable*” OR “social media*” OR “social network*” OR “e-counsel*” OR “ecounsel*” OR “palmtop*” OR “telephone*” OR “WhatsApp” OR “Twitter” OR “Facebook” OR “Instagram” OR “forum” OR “chat*” OR “virtual reality*” OR “virtual-reality*” OR “avatar*” OR “Conversational agent*” OR “virtual coach” OR “virtual agent*” OR “embodied agent*” OR “avatar*” OR “relational agent*” OR “interactive agent*” OR “virtual character*” OR “virtual human*” OR “virtual assistant*”) OR AB(“Internet*” OR “Web*” OR “Online*” OR “tele*” OR “electronic*” OR “video*” OR “device*” OR “digital*” OR “software*” OR “mobile*” OR “technolog*” OR “e-health” OR “ehealth” OR “computer*” OR “e-treat*” OR “e-therap*” OR “mhealth” OR “m-health” OR “distance counsel*” OR “cybercounsel*” OR “cyber-counsel*” OR “cyber-treat*” OR “text-messag*” OR “textmessag*” OR “text messag*” OR “SMS*” OR “texting*” OR “short message service*” OR “smartphone*” OR “cell-phone*” OR “cellphone*” OR “cellular phone*” OR “blended*” OR “handheld device*” OR “hand held device*” OR “iPad*” OR “iPhone*” OR “email*” OR “e-mail*” OR “sensor*” OR “wearable*” OR “social media*” OR “social network*” OR “e-counsel*” OR “ecounsel*” OR “palmtop*” OR OR “telephone*” OR “WhatsApp” OR “Twitter” OR “Facebook” OR “Instagram” OR “forum” OR “chat*” OR “virtual reality*” OR “virtual-reality*” OR “avatar*” OR “Conversational agent*” OR “virtual coach” OR “virtual agent*” OR “embodied agent*” OR “avatar*” OR “relational agent*” OR “interactive agent*” OR “virtual character*” OR “virtual human*” OR “virtual assistant*”) OR KW(“Internet*” OR “Web*” OR “Online*” OR “tele*” OR “electronic*” OR “video*” OR “device*” OR “digital*” OR “software*” OR “mobile*” OR “technolog*” OR “e-health” OR “ehealth” OR “computer*” OR “e-treat*” OR “e-therap*” OR “mhealth” OR “m-health” OR “distance counsel*” OR “cybercounsel*” OR “cyber-counsel*” OR “cyber-treat*” OR “text-messag*” OR “textmessag*” OR “text messag*” OR “SMS*” OR “texting*” OR “short message service*” OR “smartphone*” OR “cell-phone*” OR “cellphone*” OR “cellular phone*” OR “blended*” OR “handheld device*” OR “hand held device*” OR “iPad*” OR “iPhone*” OR “email*” OR “e-mail*” OR “sensor*” OR “wearable*” OR “social media*” OR “social network*” OR “e-counsel*” OR “ecounsel*” OR “palmtop*” OR OR “telephone*” OR “WhatsApp” OR “Twitter” OR “Facebook” OR “Instagram” OR “forum” OR “chat*” OR “virtual reality*” OR “virtual-reality*” OR “avatar*” OR “Conversational agent*” OR “virtual coach” OR “virtual agent*” OR “embodied agent*” OR “avatar*” OR “relational agent*” OR “interactive agent*” OR “virtual character*” OR “virtual human*” OR “virtual assistant*”)) AND (DE “Asthma” OR DE “Chronic obstructive pulmonary disease” OR DE “Pulmonary Emphysema” OR TX(“Asthma*” OR “COPD” OR “COAD” OR “chronic obstructive*” OR “chronic airflow obstruct*” OR “emphysema*” OR “chronic bronchitis” OR “chronic airway obstruct*” OR “obstructive pulmonary disease*” OR “obstructive respiratory disease*” OR “obstructive respiratory tract disease”)

Filters:

- Publication Year: 2000 - 2021

Pubmed

("Treatment Adherence and Compliance"[Mesh:NoExp] OR "Patient Compliance"[Mesh] OR “Patient Dropouts”[Mesh] OR fidelity[tiab] OR complian*[tiab] OR “non-complian*”[tiab] OR noncomplian*[tiab] OR adheren*[tiab] OR “non-adheren*”[tiab] OR nonadheren*[tiab] OR dropout*[tiab] OR “drop-out*”[tiab] OR “no-show*”[tiab] OR noshow*[tiab] OR attend*[tiab] OR “non-attend*”[tiab] OR nonattend*[tiab] OR absence*[tiab] OR absent*[tiab] OR “non-appear*”[tiab] OR nonappear*[tiab]) AND ("Telemedicine"[Mesh] OR "Mobile Applications"[Mesh] OR "Social Media"[Mesh] OR "Therapy, Computer-Assisted"[Mesh:NoExp] OR "Drug Therapy, Computer-Assisted"[Mesh:NoExp] OR "Telecommunications"[Mesh:NoExp] OR "Electronic Mail"[Mesh] OR "Videoconferencing"[Mesh] OR "Cell Phone"[Mesh] OR "Distance Counseling"[Mesh] OR “Wearable Electronic Devices”[Mesh] OR “virtual reality”[Mesh] OR internet*[tiab] OR web[tiab] OR “web-based*”[tiab] OR webbased*[tiab] OR online*[tiab] OR computer*[tiab] OR electronic*[tiab] OR digital*[tiab] OR ehealth[tiab] OR “e-health”[tiab] OR “e-treat*”[tiab] OR “e-therap*”[tiab] OR mhealth[tiab] OR “m-health”[tiab] OR “distance counsel*”[tiab] OR cybercounsel*[tiab] OR “cyber-counsel*”[tiab] OR “text-messag*”[tiab] OR textmessag*[tiab] OR SMS[tiab] OR texting*[tiab] OR “short message service*”[tiab] OR mobile*[tiab] OR smartphone*[tiab] OR “cell-phone*”[tiab] OR cellphone*[tiab] OR “cellular phone*”[tiab] OR blended*[tiab] OR “software app*”[tiab] OR “handheld device*”[tiab] OR “hand held device*”[tiab] OR iPad*[tiab] OR iPhone*[tiab] OR email*[tiab] OR “e-mail*”[tiab] OR sensor*[tiab] OR wearable*[tiab] OR monitoring[tiab] OR “social media*”[tiab] OR “social network*”[tiab] OR “e-counsel*”[tiab] OR ecounsel*[tiab] OR palmtop*[tiab] OR telephone*[tiab] OR WhatsApp[tiab] OR Twitter[tiab] OR Facebook[tiab] OR Instagram[tiab] OR forum[tiab] OR chat*[tiab] OR “virtual reality*”[tiab] OR avatar*[tiab] OR “Conversational agent*”[tiab] OR “virtual coach*”[tiab] OR “virtual agent*”[tiab] OR “embodied agent*”[tiab] OR “relational agent*”[tiab] OR “interactive agent*”[tiab] OR “virtual character*”[tiab] OR “virtual human*”[tiab] OR “virtual assistant*”[tiab] OR “tele-health”[tiab] OR telehealth[tiab] OR “tele-medicine”[tiab] OR telemedicine[tiab] OR “tele-care”[tiab] OR telecare[tiab] OR “tele-psychiatry”[tiab] OR telepsychiatry[tiab] OR “tele-guid*”[tiab] OR teleguid*[tiab] OR “tele-based”[tiab] OR “tele-deliver*”[tiab] OR teledeliver*[tiab] OR “tele-treat*”[tiab] OR teletreat*[tiab] OR “tele-therap*”[tiab] OR telethera*[tiab] OR “tele-intervention*”[tiab] OR “tele-counsel*”[tiab] OR telecounsel*[tiab] OR “tele-assist*”[tiab] OR teleprevent*[tiab] OR “tele-conferenc*”[tiab] OR teleconferenc*[tiab] OR “tele-monit*”[tiab] OR telemonit*[tiab] OR “tele-communicat*”[tiab] OR telecommunicat*[tiab] OR “tele-application*”[tiab] OR “tele-consult*”[tiab] OR teleconsult*[tiab] OR “video-guid*”[tiab] OR videoguid*[tiab] OR “video-mediated”[tiab] OR “video-based”[tiab] OR videobased[tiab] OR “video-deliver*”[tiab] OR “video-treat*”[tiab] OR “video-therap*”[tiab] OR videotherap*[tiab] OR “video-intervention*”[tiab] OR “video-counsel*”[tiab] OR “video-assist*”[tiab] OR “video-conferenc*”[tiab] OR videoconferenc*[tiab] OR “video-monit*”[tiab] OR videomonit*[tiab] OR “video-communicat*”[tiab] OR videocommunicat*[tiab] OR “video-remind*”[tiab] OR “video-administered*”[tiab] OR “video-aided”[tiab] OR “video-application*”[tiab] OR “video-consult*”[tiab] OR videoconsult*[tiab] OR “video-enabled”[tiab]) AND (“Asthma”[Mesh] OR “Pulmonary Disease, Chronic Obstructive"[Mesh] OR Asthma*[tiab] OR COPD[tiab] OR COAD[tiab] OR “chronic obstructive*”[tiab] OR “chronic airflow obstruct*”[tiab] OR emphysema*[tiab] OR “chronic bronchitis”[tiab] OR “chronic airway obstruct*”[tiab] OR “obstructive pulmonary disease*”[tiab] OR “obstructive respiratory disease*”[tiab] OR “obstructive respiratory tract disease*”[tiab])

Filters:

- Publication Year: March 2000 - 2021

Embase.com

('patient compliance'/exp OR 'adherence'/exp OR 'dropouts'/exp OR 'patient dropout'/exp OR ‘patient attendance’/exp OR fidelity:ab,ti,kw OR complian*:ab,ti,kw OR ‘non-complian*’:ab,ti,kw OR noncomplian*:ab,ti,kw OR adheren*:ab,ti,kw OR ‘non-adheren*’:ab,ti,kw OR nonadheren*:ab,ti,kw OR dropout*:ab,ti,kw OR ‘drop-out*’:ab,ti,kw OR ‘no-show*’:ab,ti,kw OR noshow*:ab,ti,kw OR attend*:ab,ti,kw OR ‘non-attend*’:ab,ti,kw OR nonattend*:ab,ti,kw OR absence*:ab,ti,kw OR absent*:ab,ti,kw OR ‘non-appear*’:ab,ti,kw OR nonappear*:ab,ti,kw) AND (‘telemedicine’/exp OR ‘telehealth’/exp OR ‘e-mail’/exp OR ‘mobile phone’/exp OR ‘social media’/exp OR ‘teleconference’/exp OR ‘text messaging’/exp OR ‘videoconferencing’/exp OR ‘mobile application’/exp OR ‘e-counseling’/exp OR ‘digital technology’/exp OR ‘mobile device’/exp OR ‘iphone’/exp OR ‘ipad’/exp OR 'computer assisted therapy'/de OR ‘monitoring’/exp OR ‘personal digital assistant’/exp OR ‘wearable sensor’/exp OR ‘wearable device’/exp OR ‘wearable technology’/exp OR ‘virtual reality’/exp OR ‘facebook’/exp OR ‘twitter’/exp OR internet*:ab,ti,kw OR web:ab,ti,kw OR ‘web-based*’:ab,ti,kw OR webbased*:ab,ti,kw OR online*:ab,ti,kw OR computer*:ab,ti,kw OR electronic*:ab,ti,kw OR digital*:ab,ti,kw OR ehealth:ab,ti,kw OR ‘e-health’:ab,ti,kw OR ‘e-treat*’:ab,ti,kw OR ‘e-therap*’:ab,ti,kw OR mhealth:ab,ti,kw OR ‘m-health’:ab,ti,kw OR ‘distance counsel*’:ab,ti,kw OR cybercounsel*:ab,ti,kw OR ‘cyber-counsel*’:ab,ti,kw OR ‘text-messag*’:ab,ti,kw OR textmessag*:ab,ti,kw OR SMS:ab,ti,kw OR texting*:ab,ti,kw OR ‘short message service*’:ab,ti,kw OR mobile*:ab,ti,kw OR smartphone*:ab,ti,kw OR ‘cell-phone*’:ab,ti,kw OR cellphone*:ab,ti,kw OR ‘cellular phone*’:ab,ti,kw OR blended*:ab,ti,kw OR ‘software app*’:ab,ti,kw OR ‘handheld device*’:ab,ti,kw OR ‘hand held device*’:ab,ti,kw OR iPad*:ab,ti,kw OR iPhone*:ab,ti,kw OR email*:ab,ti,kw OR ‘e-mail*’:ab,ti,kw OR sensor*:ab,ti,kw OR wearable*:ab,ti,kw OR monitoring:ab,ti,kw OR ‘social media*’:ab,ti,kw OR ‘social network*’:ab,ti,kw OR ‘e-counsel*’:ab,ti,kw OR ecounsel*:ab,ti,kw OR palmtop*:ab,ti,kw OR telephone*:ab,ti,kw OR WhatsApp:ab,ti,kw OR Twitter:ab,ti,kw OR Facebook:ab,ti,kw OR Instagram:ab,ti,kw OR forum:ab,ti,kw OR chat*:ab,ti,kw OR ‘virtual reality*’:ab,ti,kw OR avatar*:ab,ti,kw OR ‘Conversational agent*’:ab,ti,kw OR ‘virtual coach*’:ab,ti,kw OR ‘virtual agent*’:ab,ti,kw OR ‘embodied agent*’:ab,ti,kw OR ‘relational agent*’:ab,ti,kw OR ‘interactive agent*’:ab,ti,kw OR ‘virtual character*’:ab,ti,kw OR ‘virtual human*’:ab,ti,kw OR ‘virtual assistant*’:ab,ti,kw OR ‘tele-health’:ab,ti,kw OR telehealth:ab,ti,kw OR ‘tele-medicine’:ab,ti,kw OR telemedicine:ab,ti,kw OR ‘tele-care’:ab,ti,kw OR telecare:ab,ti,kw OR ‘tele-psychiatry’:ab,ti,kw OR telepsychiatry:ab,ti,kw OR ‘tele-guid*’:ab,ti,kw OR teleguid*:ab,ti,kw OR ‘tele-based’:ab,ti,kw OR ‘tele-deliver*’:ab,ti,kw OR teledeliver*:ab,ti,kw OR ‘tele-treat*’:ab,ti,kw OR teletreat*:ab,ti,kw OR ‘tele-therap*’:ab,ti,kw OR telethera*:ab,ti,kw OR ‘tele-intervention*’:ab,ti,kw OR ‘tele-counsel*’:ab,ti,kw OR telecounsel*:ab,ti,kw OR ‘tele-assist*’:ab,ti,kw OR teleprevent*:ab,ti,kw OR ‘tele-conferenc*’:ab,ti,kw OR teleconferenc*:ab,ti,kw OR ‘tele-monit*’:ab,ti,kw OR telemonit*:ab,ti,kw OR ‘tele-communicat*’:ab,ti,kw OR telecommunicat*:ab,ti,kw OR ‘tele-application*’:ab,ti,kw OR ‘tele-consult*’:ab,ti,kw OR teleconsult*:ab,ti,kw OR ‘video-guid*’:ab,ti,kw OR videoguid*:ab,ti,kw OR ‘video-mediated’:ab,ti,kw OR ‘video-based’:ab,ti,kw OR videobased:ab,ti,kw OR ‘video-deliver*’:ab,ti,kw OR ‘video-treat*’:ab,ti,kw OR ‘video-therap*’:ab,ti,kw OR videotherap*:ab,ti,kw OR ‘video-intervention*’:ab,ti,kw OR ‘video-counsel*’:ab,ti,kw OR ‘video-assist*’:ab,ti,kw OR ‘video-conferenc*’:ab,ti,kw OR videoconferenc*:ab,ti,kw OR ‘video-monit*’:ab,ti,kw OR videomonit*:ab,ti,kw OR ‘video-communicat*’:ab,ti,kw OR videocommunicat*:ab,ti,kw OR ‘video-remind*’:ab,ti,kw OR ‘video-administered*’:ab,ti,kw OR ‘video-aided’:ab,ti,kw OR ‘video-application*’:ab,ti,kw OR ‘video-consult*’:ab,ti,kw OR videoconsult*:ab,ti,kw OR ‘video-enabled’:ab,ti,kw) AND (‘asthma’/exp OR ‘chronic bronchitis’/exp OR ‘chronic obstructive lung disease’/exp OR Asthma*:ab,ti,kw OR COPD:ab,ti,kw OR COAD:ab,ti,kw OR ‘chronic obstructive*’:ab,ti,kw OR ‘chronic airflow obstruct*’:ab,ti,kw OR emphysema*:ab,ti,kw OR ‘chronic bronchitis’:ab,ti,kw OR ‘chronic airway obstruct*’:ab,ti,kw OR ‘obstructive pulmonary disease*’:ab,ti,kw OR ‘obstructive respiratory disease*’:ab,ti,kw OR ‘obstructive respiratory tract disease*’:ab,ti,kw) AND ([article]/lim OR [article in press]/lim OR [editorial]/lim OR [letter]/lim OR [review]/lim)

Filters:

- Publication Year: 2000-2021
- Article type: article in press, article, editorial, review

Cochrane library (Wiley)

([mh “patient compliance”] OR [mh “treatment adherence and compliance”] OR [mh “Patient dropouts”] OR (fidelity OR complian* OR “non-compliance” OR “non-compliant” OR noncomplian* OR adheren* OR “non-adherence” OR nonadheren* OR dropout* OR “drop-out” OR “drop-outs” OR “no-show” OR noshow* OR attend* OR “non-attendence” OR nonattend* OR absence* OR absent* OR “non-appearence” OR nonappear* ):ti,ab,kw) AND ([mh "Telemedicine"] OR [mh "Mobile Applications"] OR [mh "Social Media"] OR [mh "Therapy, Computer-Assisted"] OR [mh "Drug Therapy, Computer-Assisted"] OR [mh "Telecommunications"] OR [mh "Electronic Mail"] OR [mh "Videoconferencing"] OR [mh "Cell Phone"] OR [mh "Distance Counseling"] OR [mh “Wearable Electronic Devices”] OR [mh “virtual reality”] OR (internet* OR web OR “web-based” OR webbased* OR online* OR computer* OR electronic* OR digital* OR ehealth OR “e-health” OR “e-treatment” OR “e-therapy” OR mhealth OR “m-health” OR “distance counseling” OR cybercounsel* OR “cyber-counseling” OR “text-message” OR textmessag* OR SMS OR texting* OR “short message service” OR mobile* OR smartphone* OR “cell-phone” OR cellphone* OR “cellular phone” OR blended* OR “software app” OR “handheld device” OR “hand held device” OR iPad* OR iPhone* OR email* OR “e-mail” OR sensor* OR wearable* OR monitoring OR “social media” OR “social network” OR “e-counseling” OR ecounsel* OR palmtop* OR telephone* OR WhatsApp OR Twitter OR Facebook OR Instagram OR forum OR chat* OR “virtual reality” OR avatar* OR “Conversational agent” OR “virtual coach” OR “virtual agent” OR “embodied agent” OR “relational agent” OR “interactive agent” OR “virtual character” OR “virtual human” OR “virtual assistant” OR “tele-health” OR telehealth OR “tele-medicine” OR telemedicine OR “tele-care” OR telecare OR “tele-psychiatry” OR telepsychiatry OR “tele-guide” OR teleguid* OR “tele-based” OR “tele-deliver” OR teledeliver* OR “tele-treatment” OR teletreat* OR “tele-therapy” OR telethera* OR “tele-intervention” OR “tele-counseling” OR telecounsel* OR “tele-assistant” OR teleprevent* OR “tele-conference” OR teleconferenc* OR “tele-monitor” OR telemonit* OR “tele-communication” OR telecommunicat* OR “tele-application” OR “tele-consult” OR teleconsult* OR “video-guide” OR videoguid* OR “video-mediated” OR “video-based” OR videobased OR “video-deliver” OR “video-treatment” OR “video-therapy” OR videotherap* OR “video-intervention” OR “video-counsel” OR “video-assistant” OR “video-conference” OR videoconferenc* OR “video-monitor” OR videomonit* OR “video-communication” OR videocommunicat* OR “video-reminder” OR “video-administered” OR “video-aided” OR “video-application” OR “video-consult” OR videoconsult* OR “video-enabled”):ti,ab,kw) AND ([mh Asthma] OR [mh “Pulmonary Disease, Chronic Obstructive"] OR (Asthma* OR COPD OR COAD OR “chronic obstructive” OR “chronic airflow obstruction” OR emphysema* OR “chronic bronchitis” OR “chronic airway obstruction” OR “obstructive pulmonary disease” OR “obstructive respiratory disease” OR “obstructive respiratory tract disease”):ti,ab,kw)

Filters:

- Publication Year: March 2000 – 2021
