# Supplementary material for: Effectiveness of eHealth Interventions in Improving Medication Adherence for Patients With Chronic Obstructive Pulmonary Disease or Asthma: Systematic Review
Source: J Med Internet Res. 2021 Jul 27;23(7):e29475. doi: 10.2196/29475 (PMC8403699; doi:10.2196/29475)
Supplement: Multimedia Appendix 3 [file jmir_v23i7e29475_app3.docx]

|  | **Country** | **Primary or secondary outcome** | **Type of intervention** | **Technology** | **Comparison** | ***N*** | **Age**  **(*M±SD*)** | **Gender**  **(% females)** | **Duration**  **(months)** |
| --- | --- | --- | --- | --- | --- | --- | --- | --- | --- |
| **COPD** |  |  |  |  |  |  |  |  |  |
| Farmer, 2017  [26] | UK | Secondary | Daily symptom monitoring and self-support modules | Internet-based tablet | CAU: information leaflets with personalized information on their condition, medication use and self-management plan. | TG: 110  CG: 56 | TG: 69.8(9.1)  CG: 69.8(10.6) | TG: 38.2  CG: 39.3 | 12 |
| Garcia-Aymerich, 2007 [22] | Spain | Primary | Integrated care | Telephone | CAU: pharmacological prescriptions at discharge | TG: 44  CG: 69 | 73.0(0.8)^*^ | 14.0^*^ | 12 |
| Pinnock, 2013  [27] | UK | Secondary | Tele-monitoring | Telephone | CAU: clinical care based on (inter)national guideline recommendations | TG: 128  CG: 128 | TG: 69.4(8.8)  CG: 68.4(8.4) | TG: 59.0  CG: 51.0 | 12 |
| Wei, 2014 [23] | China | Primary | Education and counseling | Telephone | CAU: general counseling | TG: 58  CG: 59 | TG: 65.2(8.1)  CG: 63.9(6.2) | TG: 34.5  CG: 32.2 | 6 |
| Broadbent, 2018 [24] | NZ | Secondary | Tele-monitoring and medication reminders | Internet-linked robot | CAU: referral for rehabilitation and as-needed contact with respiratory physiotherapists and other clinicians | TG: 30  CG: 30 | TG: 70.6(10.3)  CG: 69.1(9.9) | TG: 63  CG: 60 | 4 |
| To, 2020 [25] | China | Primary | Tele-monitoring | Telephone | CAU: FTF general counseling | TG: 15  CG: 15 | TG: 75.6(5.0)  CG: 75.9(4.7) | TG: 6.7  CG:0 | 2 |
| **Asthma** |  |  |  |  |  |  |  |  |  |
| Bender, 2010 [285] | US | Primary | Monitoring, medication reminders, and education | Interactive Voice Response | Inhaler use tracking | TG: 25  TG: 25 | TG: 39.6(12.8)  CG: 43.5(14.2) | TG: 60.0  CG: 68.0 | 2.5 |
| Lv, 2012 [29] | China | Secondary | Asthma management reminders | Short Message Service | CG1; verbal asthma education and traditional care  CG2; asthma education | TG: 50  CG1: 50  CG2: 50 | TG: 36.2(11.1)  CG1: 37.7(11.9)  CG2: 41.1(12.1) | TG: 33.3  CG1: 48.1  CG2: 50.0 | 3 |
| Rasmussen, 2005 [34] | DK | Secondary | Monitoring and management | Internet-based tool | CG1; monitoring and treatment by specialist  CG2; monitoring and treatment by GP | TG: 100  CG1: 100  CG2: 100 | TG: 28(18-44)^‡^  CG1: 30(19-45)^‡^  CG2: 30(20-45)^‡^ | TG: 68.2  CG1: 65.9  CG2: 72.5 | 6 |
| Strandbygaard, 2010 [30] | DK | Primary + secondary | Medication reminders | Short Message Service | CAU: asthma and inhaler use education | TG: 14  CG: 12 | 32.2^*^ | 46.2^*^ | 3 |
| Vollmer, 2011 [31] | US | Primary | Medication refill reminders | Interactive Voice Response | CAU: not specified | TG: 7033  CG: 7031 | 53.6(15.3) ^*^ | 66.2^*^ | 18 |
| Young, 2012 [32] | US | Primary | Pharmacist consultations | Telephone | CAU: mail receipt of a prescription refill with written medication use instructions | TG: 49  CG: 49 | 44.6(15.8) ^*^ | 76.5^*^ | 6 |
| Bender, 2020 [33] | US | Primary | TG1/TG2: Monitoring, asthma care management support | TG1: text/phone (Interactive Voice Response)  TG2: Email | CAU: patient outreach upon refill request | TG1: 657  TG2: 621  CG: 655 | TG1:48.5(15.90  TG2:49.7(16.2)  CG: 48.2(16.1) | TG1: 59.2  TG2: 60.0  CG: 61.1 | 6 |

^*^not specified per group, ^‡^ values represent median and ranges

CAU, Care As Usual; CG, control group; FTF, face-to-face; ICS, Inhaled Corticosteroids; LABA, Long-Acting Beta-Agonists; TG, treatment group; WLC, waitlist control;
